# Report of Case of Spinal Exsection and Removal of a Tumor from the Cord

**Published:** 1890-12

**Authors:** C. D. Roy

**Affiliations:** House Surgeon Charity Hospital, New York


### Clinical Department.

### REPORT OF CASE OF SPINAL EXSECTION AND REMOVAL OF A TUMOR FROM THE CORD.

BY C. D. ROY, A. B., M. D., HOUSE SURGEON CHARITY HOSPITAL, NEW YORK.

Cerebral and spinal surgery are now assuming such a prominent importance that all cases relating to this subject are of much value, both to the practitioner and surgeon alike.

J. L., male, age 42, was admitted into the hospital on August 30th, 1888, with complete paralysis and analgesia of lower extremities. Family and previous history were negative, while the present history was given as follows :

Three days before his admission, while at work he suddenly had an intense pain in lower part of abdomen accompanied with the sensation of a constricting girdle. At the same time he fell, his legs refusing to act, accompanied also with loss of sensation up to within two inches of umbilicus, control of bladder and rectum completely lost. Otherwise the patient felt fairly well and remained in the state of no very marked improvement for weeks. Urine began to dribble from him. Examination showed a small meatus, and a meatotomy being performed a number 30 French sound was introduced with ease. This slight surgical procedure was followed with good results, in that the patient improved considerably. By continual medical treatment an improvement could be readily seen, progressing slowly in the way of power of motion.

Oct. 6, 1889.—Voluntary motions of left leg very good, no improvement in right. Both knee jerks enormously exaggerated, analgesia of left leg, but not of right. Girdle sensation around abdomen. Involuntary urination and defecation. From this time on the patient was vacillating in his improvement.

On July 15th, 1890, the condition of the patient was as follows:

General condition fair, sits up most of the day, appetite fair. Girdle sensation has been getting worse. Twitchings of muscles, especially of left leg very marked and unable to control them. Urine drawn per catheter. Sensation lost on abdomen below the umbilicus. Motion in right leg completely lost and retained in left. Sensation fair in right leg, and lost in left. Pupils normal. At this time Dr. Peterson, Neurolgist to the Hospital, diagnosed tumor of the spinal cord and located it in the lower dorsal region. Upon consultation an operation was deemed advisable; being performed by Dr. J. E. Kelley, assisted by Drs. Lloyd and Peterson. A vertical incision was made in the median line six inches long over the four lower dorsal vertebra. The spinous processes being exposed, the incisions were carried down to the bodies of the vertebrae. The periosteum being separated, with the bone forceps, the spinous and transverse processes of the four lower dorsal vertebra were removed. The dura mater was then seized with the forceps and carefully slit up for two inches, which was afterwards extended, and immediately below it and lying on the cord was recognized a tumor of some length. This was carefully dissected off and preserved for examination. Quite a little hemorrhage occurred at this stage of the procedure which, however, was quickly stopped. The wound having now been thoroughly cleaned, four deep tension sutures were inserted between which superficial sutures brought the edges of the wound together, a drainage tube having been left in at the lower end. Antiseptic dressings were applied and the patient transferred to the ward in a good condition. On the third day the temperature rose as high as 102, but was immediately checked by an antipyretic and no further rise experienced. From this time forward the patient has gradually improved and at this writing the following is his condition: There is no girdle sensation; feeling has returned; has voluntary evacuations; with the aid of a stick, can walk across the room, and his general condition is much improved. The patient hails from the old North States, and boasts with pleasure at having served under the gallant Wade Hampton. The case was one of interest, and I trust will prove so to the readers of the RECORD.

